# A gene-by-gene population genomics platform: *de novo* assembly, annotation and genealogical analysis of 108 representative *Neisseria meningitidis* genomes

**DOI:** 10.1186/1471-2164-15-1138

**Published:** 2014-12-18

**Authors:** Holly B Bratcher, Craig Corton, Keith A Jolley, Julian Parkhill, Martin CJ Maiden

**Affiliations:** Department of Zoology, University of Oxford, Oxford, UK; The Sanger Institute, Wellcome Trust Genome Campus, Hinxton, UK

**Keywords:** *Neisseria meningitidis*, *de novo* assembly, BIGSdb, Gene-by-gene analysis, cgMLST, rMLST, rST, Bacterial population genomics

## Abstract

**Background:**

Highly parallel, ‘second generation’ sequencing technologies have rapidly expanded the number of bacterial whole genome sequences available for study, permitting the emergence of the discipline of population genomics. Most of these data are publically available as unassembled short-read sequence files that require extensive processing before they can be used for analysis. The provision of data in a uniform format, which can be easily assessed for quality, linked to provenance and phenotype and used for analysis, is therefore necessary.

**Results:**

The performance of *de novo* short-read assembly followed by automatic annotation using the pubMLST.org *Neisseria* database was assessed and evaluated for 108 diverse, representative, and well-characterised *Neisseria meningitidis* isolates. High-quality sequences were obtained for >99% of known meningococcal genes among the *de novo* assembled genomes and four resequenced genomes and less than 1% of reassembled genes had sequence discrepancies or misassembled sequences. A core genome of 1600 loci, present in at least 95% of the population, was determined using the Genome Comparator tool. Genealogical relationships compatible with, but at a higher resolution than, those identified by multilocus sequence typing were obtained with core genome comparisons and ribosomal protein gene analysis which revealed a genomic structure for a number of previously described phenotypes. This unified system for cataloguing *Neisseria* genetic variation in the genome was implemented and used for multiple analyses and the data are publically available in the PubMLST *Neisseria* database.

**Conclusions:**

The *de novo* assembly, combined with automated gene-by-gene annotation, generates high quality draft genomes in which the majority of protein-encoding genes are present with high accuracy. The approach catalogues diversity efficiently, permits analyses of a single genome or multiple genome comparisons, and is a practical approach to interpreting WGS data for large bacterial population samples. The method generates novel insights into the biology of the meningococcus and improves our understanding of the whole population structure, not just disease causing lineages.

**Electronic supplementary material:**

The online version of this article (doi:10.1186/1471-2164-15-1138) contains supplementary material, which is available to authorized users.

## Background

The widespread application of parallel high-throughput ‘next generation’ sequencing (NGS) technologies has made whole genome sequence (WGS) data available for tens of thousands of bacterial isolates [1]. Increasingly, these data are publicly available only as depositions in short-read sequence archives: in December 2013 the European Bioinformatics Institute (EBI) Sequence Read Archive (SRA), contained more than 100,000 bacterial WGS records, over 90% of which comprised millions of short sequence reads each of fewer than 200 bases in length. These data represent a major resource for studies of bacterial diversity, evolution and function; however, as the throughput of genome finishing and annotation technologies has not kept pace with sequence determination, the genomes have to be reassembled to be interpreted. Typically, this is done either by mapping to a reference sequence or by *de novo* assembly to generate draft genomes comprising multiple contiguous sequences (contigs).

The approach of mapping short-read sequences to a reference sequence has been effectively used to analyse WGS data from closely related isolates in numerous studies [2–9], especially by using the data obtained to reconstruct genealogies based on phylogenetic trees. This approach has a number of limitations, including: the necessity for a high-quality reference sequence with which to make the comparison; variation in sequence not present in the reference cannot be detected; the approach is poorly scalable; analyses typically have to be re-run as new genomes are obtained; and finally, the density of sequence polymorphisms in the majority of bacterial populations is such that this approach is not feasible for the study of isolates that are not genetically closely related. The use of *de novo* assembly methods represents an alternative, more broadly applicable approach, with assemblers based on de Brujin graphing being widely used as they deal effectively with large volumes of data [10, 11] and can assemble short-read sequences of fewer than 100 bases in length into contigs that contain the majority of the genome. Further, when paired-end sequencing strategies are employed, ‘high quality draft’ bacterial genomes can be assembled [2–9, 12–14]. Once they have been assembled, these sequences can be annotated by comparisons to known genes or genome databases [15], using an approach similar to that used in multilocus sequence typing (MLST), which has been widely employed for sequence-based analyses at the population scale since 1998 [16]. The Bacterial Isolate Genome Sequence Database (BIGSdb) platform provides this functionality for WGS data [17].

*Neisseria meningitidis*, the meningococcus, is a pathogen of global significance and an informative model organism for investigating the relationship between genotype and phenotype, as it is highly diverse phenotypically and genotypically [18]. Due to the importance of the disease, the most studied meningococcal phenotype is the propensity to invade, although most episodes of meningococcal infection result in asymptomatic carriage, which typically occurs in 10-20% of the human population [19, 20]. Only a very small number of infections result in devastating and rapidly progressing disease, in the form of septicaemia, meningitis, or both. For reasons that are incompletely understood, some meningococcal genotypes are much more likely to cause invasive disease than others. Nucleotide sequence-based typing, especially MLST and antigen sequence typing (AGST), have established that these genotypes correspond to certain genealogies, known as the ‘hyperinvasive lineages’ [21]. There are a number of factors known to contribute to the hyperinvasive phenotype, particularly the possession of certain capsular polysaccharides, but species-level comparisons suggest that the majority of the pan-genome is widely shared among invasive and non-invasive genotypes. This has led to the conclusion that the ability to cause invasive disease is both polygenic and different among hyperinvasive lineages [22–24], but the determinants associated with particular lineages remain poorly defined. Comparative WGS of meningococcal isolate collections that include representative disease and carriage isolates have the potential to define the genetic differences which determine the hyper invasive phenotypes.

Here, WGS data collected by NGS technology were investigated with *de novo* assembly and population annotation to characterize 108 diverse meningococcal genomes, including the major hyperinvasive lineages observed worldwide over the last 60 years. The draft genomes were analysed for accuracy and coverage using the BIGSdb platform [17] which enabled comparison with 24 antigen and MLST typing loci previously characterised with Sanger sequencing and four finished reference genomes, cross-validating these technologies. These data established the robustness and reliability of using *de novo* draft genomes for a population-wide level of analysis for meningococcus genomes and presented a WGS description of the major hyperinvasive lineages, providing insights into their structure, evolution, and function.

## Results

### Genome assembly

Short-read sequences were assembled into draft genomes using Velvet [25] and VelvetOptimiser [26] programs, using 54 or 76 base read files. The sum total length of assembled contigs ranged from 1,975,180 bp to 2,211,536 bp and had a G + C content between 51-52%, consistent with previously finished meningococcal genomes (see Additional file 1: Table S1). Assemblies consisted of 291 to 407 contigs, with a mean of 367. The average N50, a value that represents the length at which contigs of equal or longer length contain at least 50% of the assembled sequence, across all genomes was 19,495 bp. This statistic provided an indication of the total genome coverage; however, it was not a measure of genome assembly quality. Overall, a higher k-mer setting for the assembly was associated with the higher N50 values and, within the bounds of the read length, assembled repeat regions within the genome that were under 100 bp in length.

All the *de novo* assemblies consisted of contigs terminating at repetitive sequence regions longer than the read length of 54 or 76 bases and these termination regions contained a higher read depth than the preceding regions. A change from Taq polymerase to Phusion® high-fidelity DNA polymerase affected the assembly statistics (Table 1: groups I and J), and increased the average longest contig length by 41% (Table 1: groups J and K). Improved sequencing chemistry that generated read lengths of 76 bases resulted in a 20% decrease in the average number of contigs per genome (Table 1: group K). The combined use of the new DNA polymerases and improved chemistry resulted in a decreased number of contigs and a reduction of incomplete coding sequence (CDS) of approximately 61% (Table 1: group K).

**Table 1:** Velvet assembly statistics of 108* genomes analyzed at 1605 core meningococcal loci

Multiplex group (number of libraries)	Sequence read length	Most common k-mer	Average contig count	Average N50	Average longest contig	Average genome length	Average number of core loci identified	Average number of incomplete loci found
A (12)	54	39	374	18450	66014	2088067	1603	45
B (12)	54	35	407	16252	63032	2073517	1603	39
C (12)	54	39	339	20375	76751	2087187	1602	43
D (12)	54	41	322	22022	79870	2086109	1604	34
E (12)	54	37	396	15813	59228	2108812	1602	43
F (11)	54	41	345	18364	66689	2085872	1603	40
G (12)	54	39	369	16595	61439	2107242	1602	46
H (12)	54	37	329	20557	77125	2095768	1603	46
I (11)	54	39	386	13842	53816	2085635	1604	54
J (7)	54	41	403	22586	104539	2109587	1600	37
K (7)	76	63	295	31378	122718	2143841	1604	26
**Average** ***de novo*** **Assembly Statistics**	**n/a**	**39**	**360**	**19658**	**75566**	**2097421**	**1603**	**41**

^*^The total number of genomes analysed is 120 and includes: 5 genomes sequenced a second time using 54 base reads (J) and 7 genomes sequenced a second time using 76 base reads (K).

Two genomes failed to sequence in their original groups (F and I), these genomes were subsequently rerun in group J.

The increase in read length used for multiplex group K produced larger than expected assembly improvements. A significant drop in the number of contigs and a corresponding increase in the N50 value were achieved with the relatively small 22 base read increase. Therefore, additional increased base read lengths should continue to increase the coverage of long repeat regions and decrease the number of contigs per assembly.

### Sequencing accuracy

**Figure 1:**
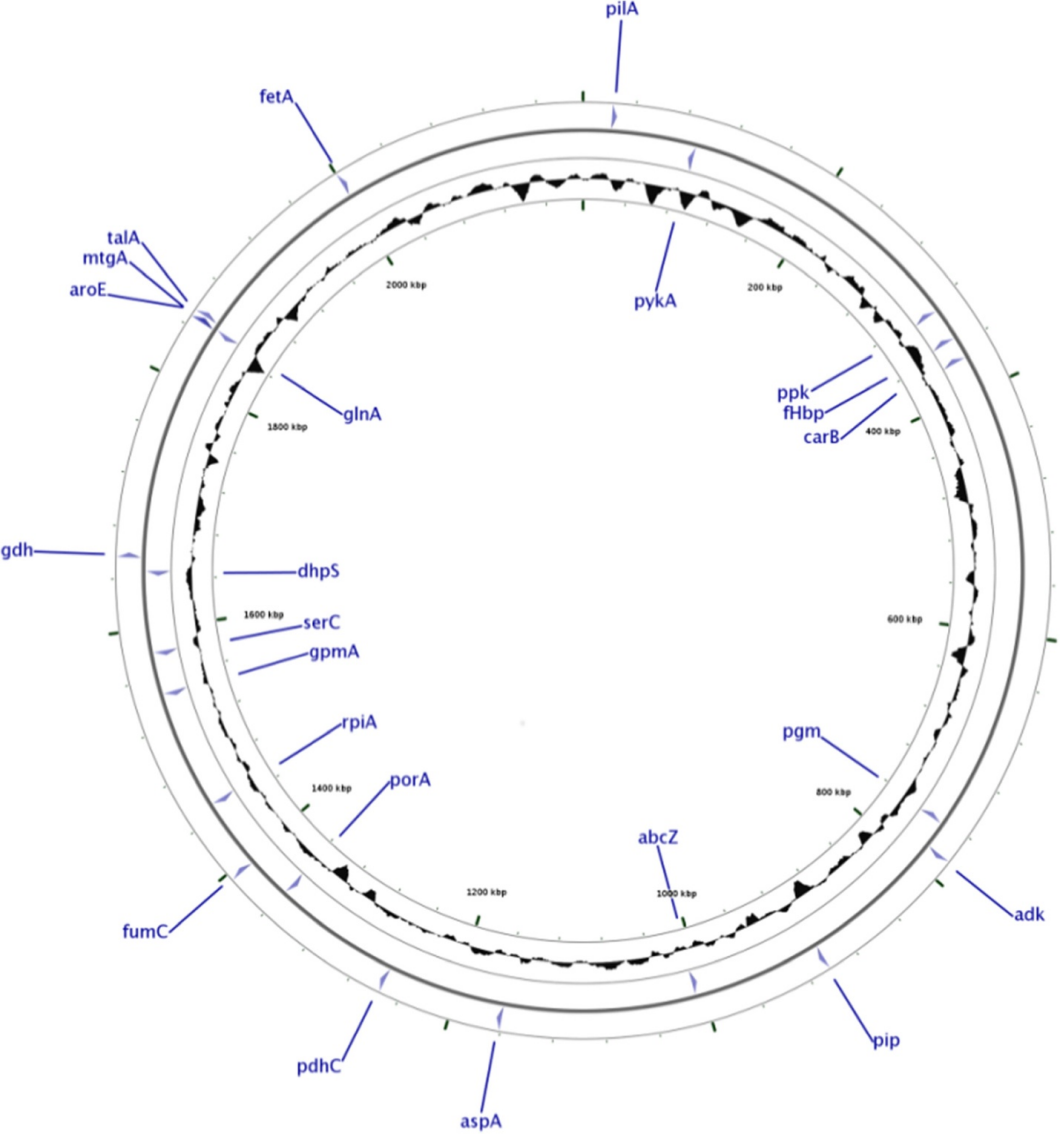
**Location of eMLST and antigen genes within the meningococcal genome.** CGView map of the *Neisseria meningitidis* reference genome, FAM18, showing the placement of the conventional and extended MLST loci and the 3 antigen genes (4 typing fragments) used to assess sequence accuracy of the *de novo* high-throughput assembly method across the genome.

The WGS assemblies were compared to previously determined dideoxy (Sanger) sequence results at the MLST [16], eMLST [27, 28], *porA VR1* and *VR2* [29], *fetA* [30] and *fHbp* [31] loci found throughout the genome (Figure 1). There were thirty-four sequence discrepancies (1.1% of CDS) between the Illumina *de novo* assembled and Sanger sequenced alleles, from 20 of the 108 genomes (Table 2). The number and distribution of sequence changes found in the resequencing experiments enabled the likely reasons for the discrepancies to be identified. In the majority of cases these could be attributed to either editing or labelling problems in the original Sanger sequencing experiments with only four instances that were a direct consequence of the assembly of the short-read sequences by the Velvet algorithm. The four MLST profiles affected by trace file editing errors maintained their original clonal complex assignment; however, their sequence type (ST) was amended to a new designation as a consequence of this work. In summary, the errors in the original Sanger experiments were due to: eleven trace file editing errors; 19 samples mislabelled during Sanger sequencing; and four occurrences of Velvet mis-assembly caused by short tandem repeat (STR) regions.

**Table 2:** **Comparison of Sanger derived MLST and AGST loci to their respective** ***de novo*** **assembled genome**

Typing locus	Original Sanger derived allele	Illumina derived allele	Retested Sanger derived allele	Number of bases, likely cause of discrepancy
**MLST**				
^*f*^ *adk*	1	10	10	9, mislabelled
^*f*^ *gdh*	8	34	34	1, editing error
^*f*^ *pdhC*	1	547	547	1, editing error
	14	60	60	1, editing error
**eMLST**				
^*f*^ *pykA*	9	5	5	35, mislabelled
^*f*^ *ppk*	3	17	17	2, editing error
	12	1	1	19, mislabelled
^*f*^ *pip*	2	1	1	6, mislabelled
	4	19	19	2, editing error
^*f*^ *dhpS*	11	87	87	10, mislabelled
	10	86	86	1, editing error
	33	42	42	1, editing error
^*f*^ *aspA*	8	78	78	6, mislabelled
^*f*^ *gpm*	7	11	11	14, mislabelled
^*f*^ *rpiA*	1	18	18	2, editing error
^*f*^ *serC*	4	56	56	2, editing error
	29	56	56	20, mislabelled
	4	5	5	8, mislabelled
	8	56	56	8, mislabelled
^*f*^ *talA*	7	20	20	3, editing error
	2	6	6	21, mislabelled
**Antigens**				
PorA VR1	7	18-1	18-1	30, mislabelled
PorA VR2	1-1	1-2	1-2	3, mislabelled
	16	3	3	30, mislabelled
	15	15-1	15-1	1, editing error
	14-1	14	14	3, repeat sequence*
fHpb	25	5	5	198, mislabelled
	39	16	16	5, mislabelled
	24	14	14	196, mislabelled
	5	22	22	5, mislabelled
	35	32	32	51, mislabelled

*repeat sequence compression during assembly; this occurred in 4 different isolates.

The four draft sequences for which finished genome sequences were available (H44/76, FAM18, Z2491, and G2136) were compared to the published closed sequences using the BIGSdb Genome Comparator tool. Sequence discrepancies were found between all four resequenced draft genomes and their respective finished reference genome. The H44/76 and G2136 reference genomes, created with Roche 454 technology and finished using capillary sequencing, had sequence differences in thirty hypothetical proteins, thirty-five annotated CDS, nine pseudogenes and five putative proteins, a total of 79 loci for these two published genomes (see Additional file 2: Table S2, sections B-E). FAM18 and Z2491 reference genomes, obtained using ABI 3700 and a combination of ABI373 and 377 respectively, had sequence discrepancies among twenty-two annotated CDS, ten pseudogenes, five putative protein sequences and fourteen hypothetical proteins; totalling 51 loci of the published CDS sequences for these genomes. The majority of these CDS affected (69.1%) had a single nucleotide change each and the remaining 30% had two or more nucleotide changes. The differences were categorized as non-synonymous or synonymous amino acid changes (see Additional file 3: Table S3). Differences caused by assembly failures (24 loci) or paralogous loci (23 loci pairs) contained cross identified reads (see Additional file 2: Table S2, sections E & F). Paralogous gene cross-identification occurred most often in CDS annotated as hypothetical proteins, a total of ten. These, plus six additional paralogous loci, were manually curated and defined using up- and down-stream sequence in order to enable the BIGSdb scanning function to correctly distinguish the divergent regions of the paralogous genes without manual curation. A list containing the identification of all CDS with sequence differences, and those loci missing in the draft genomes was generated (see Additional file 2: Table S2, section A-E).

The BIGSdb Genome Comparator tool was used to assess genome coverage of the resequenced reference genomes (Table 3a and b). The Z2491 draft genome contained 1872 of the 1876 (99.8%) CDS present in the reference genome, of which 51 (2.7%) were partial sequences, that is the locus was found at the end of a contig and therefore incompletely assembled. The four loci not identified in the draft genome belonged to four non-contiguous hypothetical proteins: NMA0440, NMA1192, NMA1307 and NMA1860, totalling 5408 nucleotides. Comparison of the FAM18 draft to the reference genome identified 1905 of 1914 (99.5%) published CDS and 107 (5.6%) were also incomplete. The nine CDS (0.5%), 21,895 bases, not found in the draft genome included three *mafB2* genes (NMC0597, NMC1790, NMC2084), a putative *frpC* pseudogene (NMC1345) and an unidentified pseudogene (NMC0296) in addition to genes encoding: a single MafB protein fragment (NMC2090), the iron-regulated FrpC protein (NMC0527), a pilus secretin (NMC0408), and putative cell-surface protein (NMC1668). The BIGSdb Genome Comparator tool identified 1967 of 1975 published CDS in the H44/76 draft genome (99.2%) and 1897 of 1904 published CDS of the G2136 draft genome (99.6%). H44/76 resequenced missing 16,716 bases that included genes encoding: three TspB family proteins (NMBH4476_0598, NMBH4476_0681, NMBH4476_1698), two iron-regulated proteins FrpA and FrpC (NMBH4476_0805, NMBH4476_1605), NlpC/p60 family protein (NMBH4476_1938), a putative Caudovirus prohead protease (NMBH4476_0570) and a hypothetical protein gene (NMBH4476_1682); and the resequenced G2136 genome was missing genes for three hypothetical proteins (NMBG2136_0443, NMBG2136_0446 and NMBG2136_0522), a membrane protein (NMBG2136_1025), a fimbral protein precursor (NMBG2136_0028) and two FrpC iron-regulated proteins (NMBG2136_0523 and NMBG2136_1306), covering 14,484 bases. All four resequenced genomes were also mapped to their respective finished genomes to look for the missing loci. In all instances the loci were present in the short-read sequence data, however read depth was low (2-5×) and therefore could not be assembled by the parameters set for the Velvet assembly.

**Table 3:** **Re-sequenced genome comparisons sequence differences identified among four re-sequenced genomes and their respective finished sequence**

a.			Missing Sequence		Failed assembly of repeat sequence tracts		Paralogous cross identification		Sequencing Discrepancy	
**Isolate**	**CDS count**	**Total number of bases**	**CDS count**	**total number of bases**	**CDS count**	**number of bases** ^**§**^	**CDS count**	**total number of bases** ^**§**^	**CDS count**	**Number of bases affected**
**Z2491**	1876	1693839	4 (0.2%)	5408	2 (0.2%)	3039	5 (0.3%)	4737	12 (0.6%)	32
**FAM18**	1914	1767562	9 (0.5%)	21895	6 (0.3%)	9070	8 (0.4%)	6990	9 (0.5%)	24
**G2136**	1904	1718346	7 (0.4%)	14484	10 (0.5%)	13125	12 (0.6%)	4974	25 (1.3%)	90
**H44/76**	1975	1784201	8 (0.4%)	16716	7 (0.4%)	14458	17 (0.9%)	4824	25 (1.3%)	76
**b.**										
**Isolate**	**Loci Present**		**Loci with identical sequence match**		**Loci with nucleotide sequence discrepancy**		**Loci that are present but incomplete**			
**Z2491**	1872 (99.8%)		1801 (96.2%)		19 (1.0%)		51 (2.7%)			
**FAM18**	1905 (99.5%)		1775 (93.2%)		23 (1.2%)		107 (5.6%)			
**G2136**	1897 (99.6%)		1757 (92.6%)		47 (2.5%)		93 (4.9%)			
**H44/76**	1967 (99.2%)		1821 (92.6%)		49 (2.5%)		97 (4.9%)			

**§** For each CDS that had either a failed assembly or paralogous cross-identification error the entire CDS length was counted as affected.

Sequence differences were identified using the BIGSdb Genome Comparator tool. All transposase CDS were removed from the analysis. The Z2491 and FAM18 were originally sequenced and finished using ABI373 and 377 or ABI 3700 methods in 2000 and 2007 respectively, and the H44/76 and G2136 genomes were originally sequenced and finished in 2011 using Roche 454 FLX and capillary-based sequencing.

While it is possible to revise the assembly parameters and recover some of the missing data in the assemblies, this would potentially be at a cost to the overall quality of the assembly by swapping specificity and sensitivity and could in fact reduce the N50 value, therefore this option was not implemented for this analysis. Technically, the foundations resulting in the underrepresentation of these regions in the subsequent sequence reads have many sources: for example GC bias affects the stability of the DNA strand which could influence the read ability or modify the probability of a fragmentation. It has been shown that optimized or PCR-free protocols reduce GC bias affects [32–35] and if these genomes were resequenced using a PCR-free approach it is possible the overall genome coverage would increase.

### Gene-by-gene annotation

All of the draft genome assemblies were annotated using a gene-by-gene approach using the BIGSdb platform as described previously [17, 36]. Each genome was scanned against defined loci contained in the PubMLST *Neisseria* sequence definition database using the default parameters (70% minimum identity; 50% minimum alignment; and a BLASTN word size of 15). Alleles previously identified were assigned an allele number automatically, in a process referred to as ‘tagging’, and new alleles were manually curated and submitted to the sequence definition database for allele number assignment. The genome data were subsequently rescanned to assign the new alleles to the respective genome in which it was found. Partially assembled loci, those found at the end of a contig, were tagged as present in the genome but flagged as incomplete. The average number of incomplete coding sequences (CDS) found per genome was 41 (see Additional file 1: Table S1). BIGSdb also identified sixteen paralogous CDS pairs, these included six recognized CDS (including two ribosomal protein genes), ten hypothetical genes and one putative lipoprotein (see Additional file 2: Table S2, section F).

### The meningococcal core genome

**Figure 2:**
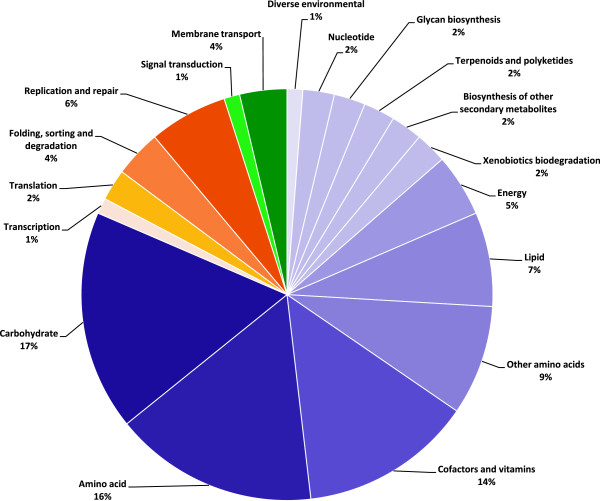
**KEGG functional and informational processing pathways identified in the meningococcal genome.** Loci from the core gene list were used to search the pathway database KEGG for functional and informational pathways. A total 537 loci of the 1605 core genes have assigned Enzyme Commission numbers (EC). The figure shows the breakdown of the genes in to three main groups and specific associated pathways: orange – genetic information processing (4 pathways, 20%), purple – metabolism (12 pathways, 73%), green – environmental information processing (2 pathways, 6%).

Comparison of the four finished genomes identified 1760 CDS (89.1%) that were present in all genomes. The list was refined by determining gene presence of the 1760 CDS in two additional finished genomes [37]. The *de novo* assembled draft genomes were then also compared to identify CDS present in 95% of the genomes, to account for the draft nature of the genomes and genes missing rarely in isolates. All genomes were unique in terms of gene content, but 1605 CDS were present in at least 95% of the isolates. These were categorised as ‘core loci’ for this dataset and were used to search the functional pathway database KEGG [38, 39] to determine the function of the gene product of each CDS (Figure 2). Only 37% of the core loci (597 in total) had an enzyme commission number (EC) assigned. Those with an assigned EC indicated the presence of two environmental processing pathways, four genetic information processing pathways and 12 metabolic functional pathways. Almost three-quarters (72%) of the EC identified genes were involved in metabolic pathways, 20% were genetic information processing pathways and 6% were identified as being involved in environmental information processing.

### Genealogical analyses

**Figure 3:**
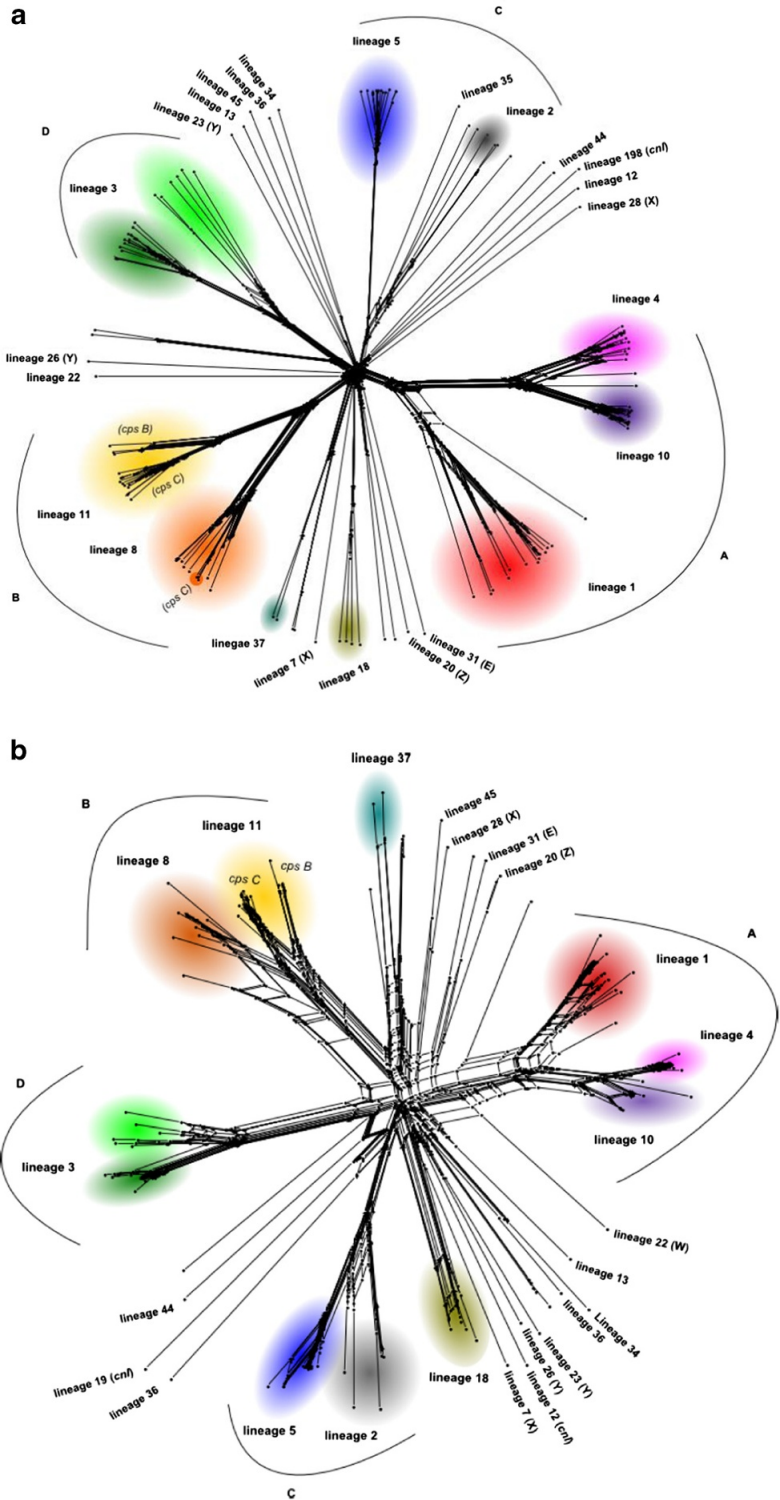
**128 representative** ***Neisseria meningitidis*** **genomes from the 20th and 21st Century.** The relationships between meningococcal isolates are represented by two datasets in which (a.) 1605 core meningococcal loci (cgMLST) or (b.) 53 ribosomal protein genes (rMLST), a subset of the 1605 core loci, are used. In both trees major phylogenetic groups are noted A-D. For cross-compatible identification, and where there are 2 or more strains per lineage are present, the major MLST derived clonal complexes (cc) are identified by colour: red – ST-1 cc, purple – ST-5 cc, pink – ST-4 cc, teal – ST-37 cc, yellow – ST-11 cc, orange – ST-8 cc, green – ST-41/44 cc, blue – ST-32 cc, grey – ST-269 cc, olive – ST-18 cc. Capsular types other than A, B, or C are noted in parentheses, accept for Lineage 11 which are labelled (cps B and cps C). Unlabelled nodes are undefined lineages and currently do not have a clonal complex association; a full list of lineage and associated clonal complex nomenclature can be found in Table 4.

Distance matrices based on the number of locus differences were calculated among the 108 isolates and 20 previously published genomes [37] with the Genome Comparator tool and represented with NeighborNet graphs for the core loci (cgMLST) and the ribosomal protein (rMLST) genes [40] (Figure 3a and b). The rMLST and cgMLST schemes clustered 101/128 (71%) of the isolates into ten distinct lineages that corresponded to the major invasive clonal complexes identified by seven-locus MLST [27] and were consistent with previously defined clades [37].

The higher resolution of rMLST and cgMLST, as opposed to the seven- or twenty- locus MLST, also resolved the substructure characteristic of lineage 3 (ST-41/44 complex). This lineage sub-structure is captured in MLST by the designation of two central genotypes that are differentially associated with invasive disease, and at the sequence type level share five of the seven MLST alleles [27, 41]. Analysis of this lineage also showed that isolates associated with the ST-41 belonged to a well-defined monophyletic lineage, while the ST-44 associated isolates were a more diverse but distinct lineage. Further exploration of this complex is necessary to more fully define the relationships within this clade and the variable pathogenic nature associated with each group. The association of capsule loci with the lineage 11 (ST-11 complex), and in lineage 8 (ST-8 complex), at the cgMLST level (1605 core genes) shows the serotype B and C associated genomes on different branches, and only lineage 11 (ST-11 complex) maintains this separation at the rMLST level (53 ribosomal genes). The remaining lineages did not have sufficient numbers to clearly differentiate capsule associations; and additional studies with larger strain collections will be required to make these associations more distinctly.

Four sets of lineage specific draft genomes, thirty-four in total, were assessed for genome coverage using one of four reference genome annotations and the BIGSdb Genome Comparator tool. Each of the four sets of *de novo* assembled genomes contained over 98% of the CDS defined by their closest reference genome. Seven isolates in the collection belong to lineage 5 (ST-32 complex) and were compared to the H44/76 reference genome. All 1976 of the H44/76 CDS were identified across the seven *de novo* assembled genomes. There was an average of 1951 CDS (98.7%) identified per genome. Seven isolates belonging to lineage 8 (ST-8 complex) were compared to the G2136 reference genome. All 1911 G2136 CDS were identified across the *de novo* assembled genomes, with an average of 1865 CDS (97.6%) found per genome. A further ten isolates belonged to lineage 11 (ST-11 complex) and were compared to the FAM18 genome sequence. The comparison identified 1912 (99.8%) of the 1915 FAM18 genome CDS across the ten genomes, with an average CDS count of 1879 CDS (98.1%) per genome. Ten isolates also belonged to the lineage 4 (ST-4 complex) and were compared to the Z2491 genome, identifying 1936 (99.9%) of 1937 CDSs across all ten genomes. Each genome had an average of 1899 CDS (98%) per genome and the only CDS not found in all ten of the lineage 4 genomes was the coenzyme A gene, *coaD*.

## Discussion

**Figure 4:**
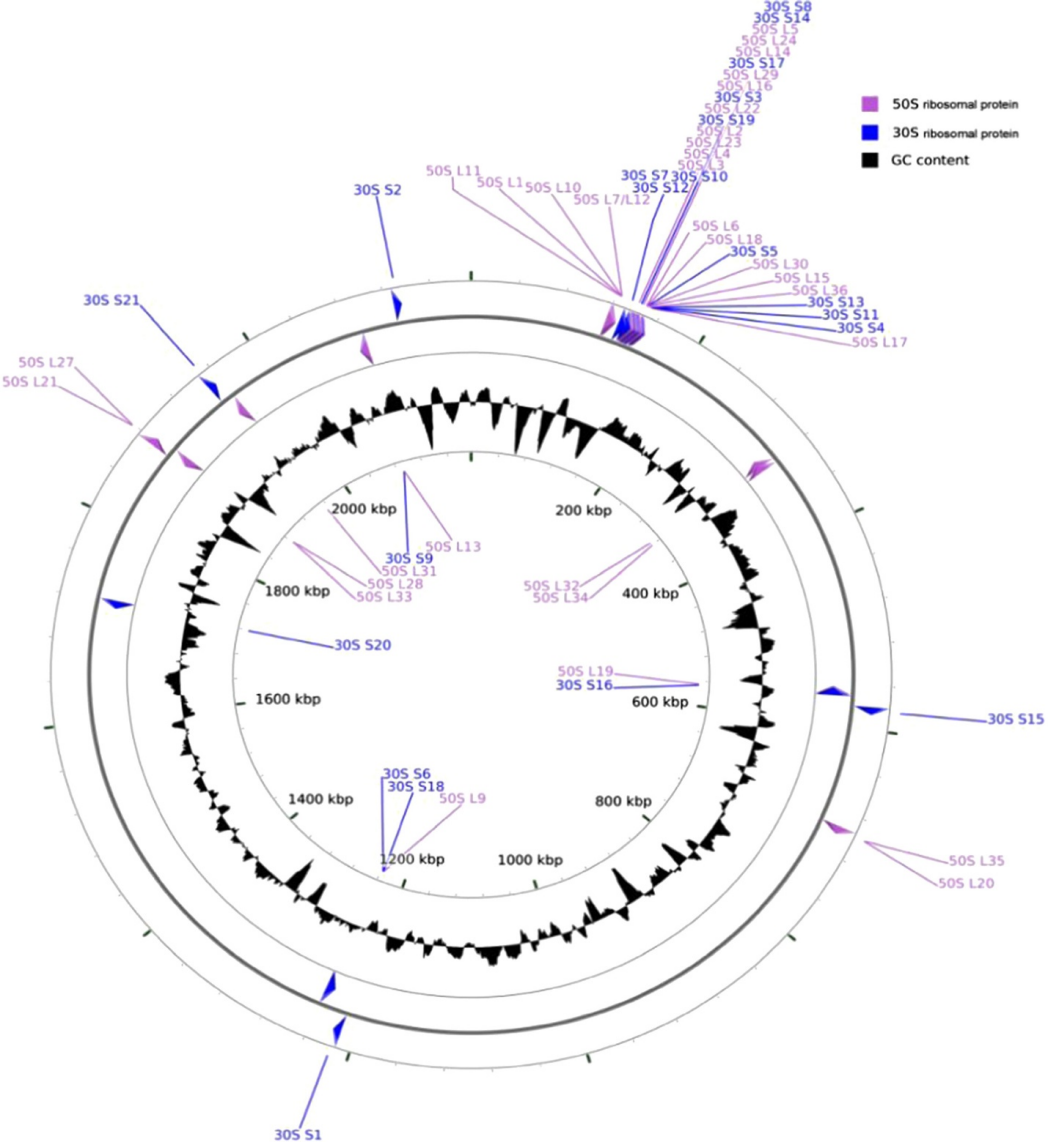
**Location of rMLST genes within the meningococcal genome.**

Exhaustive comparison of bacterial genomes, including all sources of genetic variation (i.e. individual sequence polymorphisms, insertions, deletions, and rearrangements at all scales) requires complete, closed (‘finished’) genomes. The majority, if not all, of short-read WGS data generated to date with NGS technology are incapable of meeting this ideal without extensive additional data combined with manual assembly and curation [42, 43]. There are many questions in bacterial biology, however, which can be adequately addressed with population genomic approaches that employ subsets of the genome [44], such as MLST (Figure 1), rMLST (Figure 4) and cgMLST; and for these analyses NGS datasets provide a rich source of information [15]. For such analyses to be robustly conducted, however, it is necessary to establish an analysis paradigm that interprets data consistently within known parameters of completeness and accuracy [45]. Here we demonstrate how bioinformatics tools that are freely available and widely understood can be combined to interrogate NGS data using the example of the diverse human pathogen *Neisseria meningitidis* [17]. The data and analyses are easily accessed through the PubMLST *Neisseria* website [http://pubmlst.org/neisseria/].

Although the sequence read lengths employed here were relatively short (54-76 bp) [42] and the meningococcus has a complex genome comprising many short tandem repeats (STR) and homopolymeric tracts [46–48], the Velvet algorithm was consistently capable of assembling the majority of protein coding sequences (over 1850 complete loci per genome) to extremely high levels of accuracy. Indeed, where comparable data were available for genes previously used for sequence based-typing, the majority of the discrepancies were due to errors in the editing or labelling of the specimens used in the original Sanger sequences, and the remaining, the result of STR sequence compression during assembly [49]. Once these errors had been taken in to account, the two approaches were in complete agreement. There was also very good agreement with complete reference genomes, although this depended on the read length of the short-read sequence data, with substantial improvement as read length increased. Read lengths of 100 bp, which are now routinely available, would reduce the missing data substantially [44, 50]. Data quality was also determined by the details of the chemistry and procedures used [51, 52], showing that NGS data are optimally useful when this information is deposited with them. Some coverage effects were seen, with sequences near the origin of replication consistently sequenced to a higher depth [53], than others but the genome of each assembly was adequately covered.

The BIGSdb platform accommodates sequence data derived from a particular isolate ranging from a single gene through multiple genes and contigs up to and including complete genomes [17]. The Genome Comparator tool can either use the annotations from a reference genome, which were used to compare the reference genomes with the assembled genomes, or sets of loci defined in the PubMLST sequence definition database, for which it maintains a complete catalogue of diversity described to date [15]. To enable consistent referencing each complete defined locus in the PubMLST *Neisseria* database, which can be any identifiable sequence string, was identified with a unique and arbitrary ‘NEIS’ number, which can be associated with other designations such as conventional Demeric gene names [54]. Additional loci that represent gene fragments used in typing schemes and peptide loci representing typing antigen variable regions [31, 55], are also indexed within the database. The BIGSdb ‘autotagger’ function identified and automatically annotated an average of 1899 CDS from each assembled genome, with only a small number of paralogous loci (no more than 20) in the pan-genome. The currently identified paralogous loci require additional manual annotation, they have been found to vary between the *Neisseria* species and may vary among meningococcal lineages. In conclusion, the approach can be used to analyse large numbers of WGS datasets consistently and is generally applicable for use across the bacterial domain.

Ultimately the PubMLST *Neisseria* database can be expanded, through a process of iterative gene discovery, to become a catalogue of the meningococcal ‘pan genome’ i.e. all of the genes present in the species or genus [56, 57]. This database will develop over time by a process of community annotation but, by definition, the members of the meningococcal ‘core genome’, i.e. genes present in all meningococci, will already be present. Because every bacterial isolate is potentially an unrepresentative mutant and due to the imperfect nature of NGS assemblies, the core genome cannot be simply defined as the genes present in all isolates; however, the estimate of a core genome comprising 1605 genes generated here is in good agreement with other estimates (1532-1706) which were based on substantially fewer genomes [23, 37, 58, 59]. A total 37% of the meningococcal core genes were assigned an EC number at the time of writing, indicating the magnitude of the annotation task which NGS data generates. While the membership of the core genome will be refined over time, it is unlikely to be very different from that proposed here. An updated list of meningococcal core genes will be maintained in the database.

The genealogies reconstructed with the NeighborNet algorithm using Genome Comparator data for the cgMLST and rMLST were consistent with those previously generated with MLST and a variety of other approaches [40, 60, 61]. The ribosomal genes (rMLST) and core genome (cgMLST) data provide more resolution, demonstrating that the six major hyperinvasive lineages included in this dataset cluster in to a number of larger groups [62]. Some lineages are more closely related to each other although the star phylogeny demonstrates a highly diverse and recombining population from which invasive lineages have emerged independently on several occasions [63]. As suggested from multilocus enzyme electrophoresis (MLEE) and other data [64], the serogroup A-associated lineages 1, 4 and 10 (ST-1, ST-4 and ST-5 complexes respectively) likely share a common ancestor [65], as do: lineage 8 (ST-8 complex) and lineage 11 (ST-11 complex); and lineage 5 (ST-32 complex) and lineage 2 (ST-269 complex). Lineage 3 (ST-41/44 complex) is a diverse lineage comprising both more and less invasive types. These data confirm that the invasive lineages are defined by sequence variation in the core genome, although certain members of the accessory genome, for example the capsule [66], the meningococcal disease associated island phage [67, 68], and restriction modification systems [37, 69] are differentially distributed among lineages.

To reflect the increased resolution of whole genome typing we propose the use of a lineage nomenclature (Table 4) to distinguish groupings obtained by rMLST and cgMLST from the clonal complex association identified by MLST. This nomenclature also allows for the designations of sub-lineages which our data set, and others not described here, define additional prevalent biological and phenotypic associations such as the ET-15 mutants of lineage 11. The proposal was presented to a satellite sub-group meeting of the XIX International Pathogenic *Neisseria* Conference in October 2014, which included submitters, curators and users and the proposal is under consideration for adoption by the PubMLST Management Committee.

**Table 4:** **Proposed Whole Genome Lineage Nomenclature**

WGS nomenclature	MLST nomenclature
Lineage 11 ^	ST-11 cc
Lineage 3 ^	ST-41/44 cc
Lineage 23 ^	ST-23 cc
Lineage 1	ST-1 cc
Lineage 2	ST-269 cc
Lineage 4	ST-4 cc
Lineage 5	ST-32 cc
Lineage 6	ST-60 cc
Lineage 7	ST-750 cc
Lineage 8	ST-8 cc
Lineage 9	ST-92 cc
Lineage 10	ST-5 cc
Lineage 12	ST-53 cc
Lineage 13	ST-213 cc
Lineage 14	ST-174 cc
Lineage 15	ST-1157 cc
Lineage 16	ST-116 cc
Lineage 17	ST-175 cc
Lineage 18	ST-18 cc
Lineage 19	ST-198 cc
Lineage 20	ST-103 cc
Lineage 21	ST-212 cc
Lineage 22	ST-22 cc
Lineage 24	ST-106 cc
Lineage 25	ST-162 cc
Lineage 26	ST-167 cc
Lineage 27	ST-178 cc
Lineage 28	ST-181 cc
Lineage 29	ST-226 cc
Lineage 30	ST-231 cc
Lineage 31	ST-254 cc
Lineage 32	ST-282 cc
Lineage 33	ST-292 cc
Lineage 34	ST-334 cc
Lineage 35	ST-35 cc
Lineage 36	ST-364 cc
Lineage 37	ST-37 cc
Lineage 38	ST-376 cc
Lineage 39	ST-461 cc
Lineage 40	ST-549 cc
Lineage 41	ST-865 cc
Lineage 42	ST-1117 cc
Lineage 43	ST-1136 cc
Lineage 44	ST-4821 cc
Lineage 45	ST-4240/6688 cc

**^ Distinct** sub-lineages present; proposal to use decimal based (i.e. 11.1, 11.2, etc.) system for defined sub-lineages.

To simplify and differentiate between MLST typing and whole genome based typing we propose a lineage nomenclature that is associated with defined clonal complex (cc). The data includes all PubMLST *Neisseria* database isolates.

## Conclusions

WGS data has the potential to unify studies of bacteria by providing comprehensive descriptions of genomic variation. To achieve this it is necessary to: (i) make the data available in a comprehensible way, along with information describing its completeness and accuracy; and (ii) link them to provenance and phenotype information, which describes the source of the sample and its properties, as well as the known properties of the genes identified and the deduced product. These datasets will grow in completeness and accuracy over time; however, it is also necessary for these data to be presented in a stable context, enabling even incomplete information to be explored. The approach described and validated here for the meningococcus is one way of achieving this, which employs generic, freely accessible and widely used tools. The use of the web interface within the PubMLST *Neisseria* database enables a process of community annotation whereby different members of the community can participate in the maintenance and improvement of sequence annotation and interpretation.

## Methods

### Bacterial strains and genomic DNA extraction

Genomic DNA from 108 diverse *Neisseria meningitidis* isolates was prepared from archive stocks which have been extensively characterized and previously reported [16, 27, 30, 70]; this data set includes the 107 MLST global reference collection isolates and FAM18 [16, 47]. Cultures were incubated on Columbia horse-blood ager (Oxoid) at 37°C in an atmosphere of 5% CO2 for 24 hours, sub-cultured and genomic DNA extracted using the Wizard® Genomic DNA Purification Kit (Promega).

### Illumina sequencing

Standard Illumina multiplex libraries, grouped A-K, were generated. Adapter ligated DNA was amplified by PCR using Taq or Phusion® DNA polymerase and primers from the Illumina multiplexing sample preparation oligonucleotide kit, creating up to 12 libraries per group. Before and after each of these steps DNA was simultaneously cleaned up and size selected using a 1:1 (sample:beads) ratio of Ampure beads (Beckman Coulter Genomics). Libraries were pooled in equimolar ratio and a maximum of twelve tagged, paired-end library aliquots were run per flowcell lane; every eighth lane contained the control genome, phiX 174. A standard Illumina clustering protocol was used with an additional QC step after cluster amplification. Passing flowcells were sequenced using the Illumina Genome Analyser II platform. Sequence reads have been deposited in the European Nucleotide Archive [EMBL: ERS006904 to ERS007010].

### Genome assembly

Short-read sequences were assembled using the VelvetOptimiser *de novo* short-read assembly program optimisation script using the default parameters [25, 26]. Once generated, there was no further manipulation of the assembled draft genome sequences.

### Method analysis

For each step of the process where variation or patterning, not associated with or inherent in the genome biology could be introduced, non-biological run nodes were recorded. These included notations of: date; technician; reagent lot used; manual and robotic library preparation methods including plate lane; and sequencing steps specifically noting chemistry changes, flowcell lane, number of samples per multiplex group, and the machine used.

### BIGSdb genome annotation and locus tagging

The sequence definition database was seeded using the core loci identified in finished *Neisseria meningitidis* genome annotations. The locus tag identifiers, ‘NEIS’ followed by an integer, was adopted in order to allow automated accessioning of loci as they are identified and added to the database. The NEIS, (short for ‘*Neisseria* genus’) loci list was determined using the genome annotations of FAM18, H44/76, G2136, Z2491 and MC58 and represent, notionally, the pan-genome of the meningococcus. This included the ribosomal protein loci, a sub set of the core loci which are also orthologous across all bacterial species [40]. The NEIS identifiers are linked to an alias table that contains additional locus nomenclature associated with each locus which is searchable and therefore cross compatible with various annotations; such as specific finished genome locus tags, KEGG EC or common name. The number of loci contained in the list of the NEIS locus identifiers is not static and will change as loci are curated and added to the database over time.

The draft genome sequences were queried within BIGSdb using BLAST against the sequence definition database to identify defined allelic variation. Alleles were automatically annotated and assigned with the appropriate allele number for those loci for which definitions exist, in a process referred to as ‘tagging’ while new alleles were manually curated and assigned a new allele accession number. For the gene sequences with frame shift mutations, internal stop codons, etc., the sequence was assigned an allele designation and flagged as having an internal stop codon. Any gene sequences with missing data, i.e. those at the ends of contigs, were flagged as incomplete and not assigned an allele number. Once identified the locus allelic variant was linked to the isolate metadata.

### Reference to *de novo*genome comparisons

Assembled draft genome sequences were compared to their reference genome using the BIGSdb Genome Comparator tool and assessed using the finished genome CDS sequence annotation. Genes from each genome were also compared to previously typed loci, including conventional and extended MLST loci, three antigen loci, *PorA VR1* and *VR2*, *FetA VR*, and *fHbp*, a surface antigen being explored as a vaccine candidate.

### Sanger sequencing

Sanger sequencing was performed for resolution of typing loci conflicts found between Illumina and Sanger derived sequences using a reserved sample of the DNA used for Illumina sequencing. Reserved Illumina DNA was amplified and sequenced using previously published methods and primers for conventional MLST, eMLST, *PorA VR1, PorA VR2*, or *fHbp* loci [16, 27, 29, 31]. Sanger trace files were assembled using the Staden sequence assembly package [71], and compared to the Illumina derived sequence using the MEGA5 alignment tools [72].

### Bowtie and tablet

Read depth and sequence conflicts were checked by remapping using the Bowtie short-read aligner [73]. For target sequence assessment the contig containing the typing loci was extracted from the sequence bin and used as the reference segment and the FAM18, Z2491, H44/74 and G2136 finished genomes were used for read mapping their resequenced genomes respectively. Briefly, the short-reads were converted to SAM files and mapped against the reference segment using a randomized alignment order to avoid mapping bias. Aligned .SAM files were visualized using the Tablet software package [74]. Read depth and conflicting nucleotides of interest were identified and investigated.

### Data access

Assembled contigs and annotation information can be accessed at PubMLST *Neisseria* database [http://pubmlst.org/neisseria/] using the query search, project ‘107 global collection’. Sequence reads have also been deposited in the European Nucleotide Archive (ENA) EMBL: ERS006904 to ERS007010 inclusive.

## Additional files

References to the data sets supporting the results of this article are included within the article and its 3 additional .pdf files.

## Electronic supplementary material

Additional file 1: Table S1: Velvet *de novo* assembly output statistics. (PDF 420 KB)

Additional file 2: Table S2: Loci with sequence discrepancies between the reference and resequenced genome. (PDF 399 KB)

Additional file 3: Table S3: Sequence discrepancy categories. (PDF 177 KB)
