# Supplementary material for: A gene-by-gene population genomics platform: de novo assembly, annotation and genealogical analysis of 108 representative Neisseria meningitidis genomes
Source: BMC Genomics. 2014 Dec 18;15(1):1138. doi: 10.1186/1471-2164-15-1138 (PMC4377854; doi:10.1186/1471-2164-15-1138)
Supplement: Supplementary file 1 — Additional file 1: Table S1: Velvet de novo assembly output statistics. (PDF 420 KB) [file 12864_2014_6881_MOESM1_ESM.pdf]

Additional Table 1

Velvet *de novo* assembly output statistics of 120 genomes sorted by multiplex group and assessed for 1605 core loci. 'Good' assemblies had a long k-mer value with (i) a large maximum contig length, (ii) a large N50 contig size, (iii) the smallest number of contigs and (iv) a total assembly length that closely matched that of known reference genomes.

multiplex group	Isolate	pubMLST isolate id	kmer size	number of contigs	N50 value	longest contig	total length	# core genes found	number of incomplete
**A**	F4698	120	33	395	12,600	49,668	2,060,501	1605	20
	F6124	128	31	455	11,285	45,224	2,056,609	1603	90
	H44/76	237	37	338	20,701	57,598	2,099,963	1605	0
	500	343	39	304	21,394	97,812	2,070,954	1605	30
	BZ 10	398	33	346	18,670	50,095	2,052,180	1605	51
	BZ 169	408	37	329	22,242	94,444	2,142,210	1605	33
	BZ 198	409	35	329	23,777	77,381	2,092,617	1602	36
	NG PB24	434	39	321	25,396	78,292	2,114,016	1600	40
	NG P20	436	39	305	21,082	97,812	2,066,372	1605	33
	8680	441	31	375	16,653	48,493	2,088,107	1605	50
	3906	443	35	415	15,772	54,108	2,070,442	1592	58
	1000	446	31	578	11,824	41,239	2,142,832	1600	98
**B**	153	238	33	360	14,696	56,581	2,060,468	1600	5
	297-0	442	31	551	9,690	39,840	1,975,180	1595	10
	14/1455	451	33	396	12,729	56,942	2,062,931	1605	0
	G2136	638	29	350	16,159	88,069	2,032,480	1603	2
	B6116/77	639	35	342	17,161	87,501	2,062,986	1605	0
	312 901	642	39	289	20,949	69,056	2,053,253	1604	1
	AK50	647	35	376	19,451	58,724	2,142,828	1604	1
	M-101/93	648	35	433	16,908	54,910	2,140,588	1603	2
	M40/94	650	37	309	26,096	87,419	2,134,770	1605	0
	88/03415	654	27	701	7,921	30,184	2,072,899	1605	0
	860060	657	39	375	18,036	68,810	2,109,112	1602	3
	890326	658	33	407	15,225	58,343	2,034,704	1599	6
**C**	2059001	387	43	284	25,837	99,092	2,078,511	1605	30
	SB25	640	31	456	10,361	30,130	2,044,661	1601	111
	94/155	641	33	310	18,055	91,556	2,047,907	1604	56
	204/92	645	35	418	16,437	72,963	2,128,705	1605	57
	400	646	41	327	25,497	105,386	2,120,654	1604	32
	50/94	649	39	348	22,754	77,352	2,108,496	1605	41
	N45/96	652	39	353	21,906	67,724	2,120,144	1605	36
	E32	655	37	346	21,399	86,854	2,123,723	1603	42
	E26	656	39	264	21,788	82,564	2,038,548	1590	22
	A22	659	43	326	23,475	64,764	2,141,924	1603	26
	71/94	660	39	311	18,826	87,551	2,045,624	1599	29
	860800	661	39	323	18,170	55,079	2,047,351	1597	28
**D**	7891	7	41	291	23,459	74,420	2,068,305	1604	21
	20	34	41	318	19,311	54,815	2,082,218	1605	28
	26	35	39	294	24,570	74,067	2,070,452	1605	32

	S5611	67	41	337	22,332	103,457	2,104,463	1605	39
	154	239	41	274	22,576	63,682	2,062,368	1605	27
	D8	316	37	291	23,361	112,831	2,070,336	1601	35
	NG 3/88	417	41	361	21,693	63,914	2,131,612	1603	39
	Z2491	613	41	293	23,311	99,084	2,079,826	1605	38
	AK22	643	41	341	20,443	91,975	2,077,842	1603	38
	L93/4286	644	41	326	22,290	82,476	2,080,870	1605	37
	931905	651	35	384	17,823	47,488	2,090,848	1604	34
	91/40	653	41	349	23,097	90,226	2,114,167	1603	34
**E**	243	52	39	317	17,283	77,632	2,069,890	1605	38
	CN100	90	35	316	19,064	73,069	2,130,298	1605	45
	BZ 147	403	37	474	12,070	42,975	2,102,833	1604	64
	DK 353	412	39	359	16,773	54,630	2,081,831	1601	51
	EG 328	413	37	443	18,002	58,857	2,195,602	1602	46
	EG 011	416	31	404	15,108	36,790	2,112,591	1600	53
	NG 6/88	419	37	396	14,421	69,438	2,063,651	1593	41
	NG G40	425	35	414	16,664	48,685	2,116,622	1599	32
	NG 080	430	35	397	14,987	61,702	2,109,324	1605	51
	528	445	35	525	11,108	62,642	2,113,911	1597	25
	371	466	41	347	17,599	69,495	2,088,208	1605	30
	79126	494	37	360	16,676	54,826	2,120,981	1605	42
**F**	6748	10	43	356	19,249	51,927	2,096,380	1605	19
	80049	299	35	339	15,944	53,762	2,135,885	1603	36
	D1	314	39	311	19,986	69,585	2,067,345	1605	41
	90/18311	391	41	310	20,580	54,431	2,067,022	1603	40
	BZ 163	407	39	306	18,208	69,617	2,112,365	1604	37
	NG F26	420	35	323	19,217	87,452	2,058,744	1604	40
	NG H41	422	39	434	14,287	69,665	2,087,496	1598	68
	NG E28	426	39	386	17,364	61,923	2,115,078	1596	49
	BRAZ10	468	41	343	18,376	54,425	2,067,672	1605	40
	MA-5756	507	41	340	17,733	49,614	2,067,675	1605	43
	92001	597	41	302	19,796	50,174	2,080,136	1605	28
**G**	A4/M1027	1	39	364	15,404	50,093	2,069,108	1603	66
	139M	13	35	436	11,994	62,369	2,135,812	1605	31
	H1964	210	35	314	15,831	59,026	2,070,317	1605	43
	BZ 232	410	39	383	19,322	62,152	2,131,285	1598	28
	DK 24	411	39	383	19,327	75,997	2,160,627	1600	36
	EG 327	414	37	406	13,373	36,942	2,079,945	1600	41
	NG H15	421	39	393	15,387	57,400	2,097,789	1605	58
	NG E30	427	37	323	16,946	51,511	2,053,239	1603	43
	NG H36	428	41	342	19,230	66,445	2,149,056	1604	49
	SWZ107	444	39	381	16,136	58,145	2,124,785	1593	41
	690	467	35	296	19,054	74,942	2,065,096	1604	40
	79128	492	37	406	17,138	82,248	2,149,843	1601	70
**H**	S3131	19	41	264	24,737	96,136	2,074,237	1605	35
	S4355	24	41	319	22,947	89,288	2,089,442	1605	36

	255	46	39	302	20,445	74,202	2,067,387	1605	43
	254	64	37	358	15,763	69,510	2,069,498	1604	56
	IAL2229	84	37	272	21,515	96,122	2,064,262	1605	32
	196/87	340	37	348	20,035	77,445	2,144,814	1605	66
	38VI	349	37	303	21,991	97,811	2,064,093	1605	43
	NG 4/88	418	37	322	21,855	59,704	2,058,894	1596	45
	NG H38	423	37	369	18,573	60,656	2,129,552	1601	55
	NG E31	424	39	418	18,401	83,563	2,146,433	1598	40
	106	488	37	309	22,754	77,691	2,085,979	1605	34
	322/85	493	33	366	17,670	43,368	2,154,620	1604	69
**I**	120M	2	33	463	10,581	33,563	2,074,516	1605	72
	129	11	35	347	14,410	79,861	2,083,935	1605	42
	10	31	39	354	14,054	53,317	2,070,182	1605	51
	393	61	35	397	14,162	47,836	2,081,413	1604	55
	11-004	82	39	348	13,854	47,478	2,068,319	1604	48
	1014	160	39	363	14,158	51,115	2,067,144	1605	50
	F1576	344	37	335	15,602	38,728	2,057,731	1605	47
	BZ 83	400	35	373	14,417	56,792	2,092,840	1603	50
	BZ 133	401	39	374	17,758	63,829	2,106,158	1605	42
	EG 329	415	35	439	12,338	45,158	2,136,007	1604	69
	NG144/82	431	37	451	10,926	74,300	2,103,741	1604	68
**J**	FAM18	4	41	466	16,865	69,539	2,067,451	1605	70
	139M	13	43	406	23,609	113,337	2,155,870	1605	31
	M597	369	41	344	21,063	111,183	2,068,928	1604	42
	DK 24	411	43	397	23,918	96,694	2,172,284	1600	36
	NG G40	425	37	437	23,891	103,915	2,120,214	1599	32
	528	445	41	444	23,521	100,197	2,139,911	1597	25
	E26	656	41	324	26,136	136,907	2,042,452	1590	22
**K**	6748	10	61	296	40,825	175,414	2,144,958	1605	19
	139M	13	57	294	26,154	149,689	2,141,819	1605	31
	F4698	120	63	243	29,907	81,537	2,132,393	1605	20
	BZ 169	408	63	269	36,513	99,534	2,211,536	1605	33
	297-0	442	63	289	29,365	102,428	2,065,716	1595	27
	14/1455	451	67	374	20,999	92,289	2,137,543	1605	24
	88/03415	654	63	297	35,880	158,137	2,172,921	1605	30
**mean assembly values for all multiplex groups**			**n/a**	**361**	**19,103**	**72,140**	**2,095,268**	**1603**	**38**
