# Supplementary material for: A gene-by-gene population genomics platform: de novo assembly, annotation and genealogical analysis of 108 representative Neisseria meningitidis genomes
Source: BMC Genomics. 2014 Dec 18;15(1):1138. doi: 10.1186/1471-2164-15-1138 (PMC4377854; doi:10.1186/1471-2164-15-1138)
Supplement: Supplementary file 2 — Additional file 2: Table S2: Loci with sequence discrepancies between the reference and resequenced genome. (PDF 399 KB) [file 12864_2014_6881_MOESM2_ESM.pdf]

Additional table 2 Loci with sequence discrepancies between the reference and re-sequenced genome

Comparison of finished reference to de novo assembled genome and the allelic variation found and grouped into one of five categories (A-F). Annotated loci of H44/76, Z2491, FAM18, and G2136 finished genomes were compared to their corresponding de novo resequenced genome using the BIGSdb Genome Comparator Tool. Differences were investigated using MEGA5 and Bowtie and reciprocal BLAST searches were done to identify the paralogous matches. Section (A) lists the loci in each de novo assembled genome that were unidentified and section (F) lists loci that had sequence differences but were not caused by sequencing errors, but instead paralogous loci cross-identification by BIGSdb scanning program. Section (B-D) lists the loci that have credible sequencing errors while section (E) lists loci that failed to assemble fully due to repeat sequence tracts. Loci with a reference to resequenced discrepancy are in **bold**; orthologs of each locus identified are also listed for each genome, with potential orthologs in parentheses.

**Finished genome (potential) orthologous locus tag identifier**					
**G2136**	**H44/76**	**Z2491**	**FAM18**	**BIGSdb**	Annotation [#]
**A. Loci missing in the re-sequenced, *de novo* assembled genomes**					
NMBG2136_1893	**NMBH4476_1938**	NMA0440 *	NMC1978 *	**NEIS1978**	(nlpC) p60 family protein
NMBG2136_2006	NMBH4476_2050	NMA0324	**NMC2084**	**NEIS2084**	adhesin (mafB2)
NMBG2136_0596	NMBH4476_0371	NMA2113	**NMC1790**	nca	adhesin (mafB2)
NMBG2136_1726	NMBH4476_0383	NMA2113	**NMC0597**	**NEIS0597**	adhesin (mafB2)
NMBG2136_1619	NMBH4476_1199	NMA2005	**NMC1668**	**NEIS1668**	cell-surface protein
**NMBG2136_0443**	NMBH4476_1677	(NMA0688)	NMC0450	nca	conserved hypothetical protein
**NMBG2136_0446**	NMBH4476_1682	NMA0690	NMC0450	nca	conserved hypothetical protein
**NMBG2136_0028**	NMBH4476_0024	NMA0272	NMC0003	nca	fimbrial protein precursor
-	-	**NMA0532**	-	nca	hypothetical protein
NMBG2136_0966	NMBH4476_1083	**NMA1192**	NMC0972	nca	hypothetical protein
NMBG2136_0966	NMBH4476_1083	**NMA1307**	NMC0972	nca	hypothetical protein
**NMBG2136_0522**	NMBH4476_0489	NMA0786	NMC1653	nca	hypothetical protein
(NMBG2136_0437)	**NMBH4476_1682**	NMA0690	NMC0452	nca	hypothetical protein
NMBG2136_0966	NMBH4476_1083	**NMA1860**	NMC0972	nca	hypothetical protein
NMBG2136_0523	NMBH4476_0805	NMA1626	**NMC0527**	nca	iron-regulated protein (frpC)
**NMBG2136_0523**	**NMBH4476_1605**	NMA1626	NMC0527	nca	Iron-regulated protein (frpA)
**NMBG2136_1306**	**NMBH4476_0805**	NMA1626	NMC0527	nca	Iron-regulated protein (frpC)
**NMBG2136_1025**	NMBH4476_1097	-	-	nca	membrane protein
-	-	-	**NMC0296**	nca	pseudogene
-	-	-	**NMC1345**	nca	pseudogene
-	-	**NMA0787**	-	nca	pseudogene
NMBG2136_2006	NMBH4476_2050	NMA0324	**NMC2090**	**NEIS2090**	pseudogene (mafb protein fragment)
NMBG2136_1527	**NMBH4476_0570**	NMA1914	NMC1573	nca	putative Caudovirus prohead protease (phage associated protein)
NMBG2136_0019	**NMBH4476_0598**	NMA1797	NMC1715	nca	tspB family protein
NMBG2136_0019	**NMBH4476_0681**	NMA1797	NMC0025	nca	tspB family protein
NMBG2136_0019	**NMBH4476_1698**	NMA1797	NMC1866	nca	tspB protein
NMBG2136_0407	NMBH4476_1758 *	NMA0650	**NMC0408**	**NEIS0408**	type IV pilus secretin (pilQ)
**B. Non-synonymous amino acid change**					
**NMBG2136_1781**	NMBH4476_0312	NMA2170	NMC1854	**NEIS1854**	7-cyano-7-deazaguanine reductase (queF)
NMBG2136_0481	NMBH4476_1645	**NMA0724**	NMC0484	**NEIS0484**	chromosome segregation protein
**NMBG2136_1411**	NMBH4476_0699	NMA1729	NMC1458	**NEIS1458**	conserved hypothetical protein
NMBG2136_1570	NMBH4476_0527	**NMA1952**	NMC1611	**NEIS1611**	conserved hypothetical protein
NMBG2136_0979	**NMBH4476_1172**	-	-	nca	hypothetical protein
**NMBG2136_1921**	**NMBH4476_1964**	NMA0418	NMC2001	**NEIS2001**	hypothetical protein
NMBG2136_0794	NMBH4476_1326	**NMA1073**	NMC0801	**NEIS0801**	hypothetical protein
NMBG2136_0798	NMBH4476_1319	**NMA1083**	NMC0806	nca	hypothetical protein
NMBG2136_0912	NMBH4476_1232	**NMA1135**	NMC0917	**NEIS0917**	hypothetical protein
-	**NMBH4476_0323**	-	-	nca	hypothetical protein
**NMBG2136_0274**	NMBH4476_0268	NMA2214 *	NMC0832	nca	hypothetical protein
NMBG2136_0303	NMBH4476_1858 *	**NMA0537**	NMC0306	**NEIS0306**	hypothetical protein / integral membrane protein

NMBG2136_1513	NMBH4476_0584	**NMA1897**	NMC1557	**NEIS1557**	initiation factor IF2 (infB)
NMBG2136_1372	**NMBH4476_0741**	NMA1692	**NMC1418**	**NEIS1418**	lipoprotein (nlpD)
-	-	**NMA1997**	-	nca	pseudogene
-	-	**NMA0266**	-	nca	pseudogene
-	-	**NMA0267**	-	nca	pseudogene
NMBG2136_0343	NMBH4476_1819	NMA0579	**NMC0342**	**NEIS0342**	putative prolyl endopeptidase
NMBG2136_1183	NMBH4476_0936	NMA1483	**NMC1208**	**NEIS1208**	putative transmembrane transport protein
NMBG2136_0941	**NMBH4476_0480**	NMA1167	NMC1661	**NEIS0661**	replication initiation factor
NMBG2136_0668	NMBH4476_1468	NMA0929	**NMC0672**	**NEIS0672**	threonyl-tRNA synthetase (thrS)
NMBG2136_1246	NMBH4476_1774	NMA1558	**NMC1282**	**NEIS1282**	TonB-dependent receptor protein
NMBG2136_1758	**NMBH4476_0336**	NMA2146	**NMC1829**	**NEIS1829**	tspA antigen protein
**NMBG2136_0019**	NMBH4476_0681	**NMA1797**	NMC0283	**NEIS0283**	tspB protein (part of MDA island)
**NMBG2136_1767**	NMBH4476_0327	NMA2156	NMC1839	**NEIS1839**	type 4 prepilin-like proteins leader peptide-processing enzyme (pilD)
**C. Synonymous amino acid change**					
**NMBG2136_1779**	NMBH4476_0314	NMA2168	NMC1852	**NEIS1852**	antibacterial fatty acid resistance protein B
**NMBG2136_0798**	NMBH4476_1326	NMA1073	NMC0801	**NEIS0801**	conserved hypothetical protein
**NMBG2136_0441**	NMBH4476_1679	NMA0690	NMC0448	nca	conserved hypothetical protein
**NMBG2136_1032**	NMBH4476_1090	NMA1881	NMC1048	nca	DNA transposition protein (gpB)
NMBG2136_2041	NMBH4476_2092	NMA0241	**NMC2132**	**NEIS2132**	electron transfer flavoprotein alpha-subunit (etfA)
NMBG2136_0784	**NMBH4476_1336**	NMA1063	NMC0852	**NEIS0792**	GTP-binding protein
NMBG2136_0274 *	NMBH4476_0268	**NMA2214**	NMC0832	nca	hypothetical protein
**NMBG2136_0605**	**NMBH4476_1531**	-	NMC0606	nca	hypothetical protein
NMBG2136_0443	**NMBH4476_1677**	NMA0688	NMC0450	nca	hypothetical protein
NMBG2136_0787	NMBH4476_1333	**NMA1066**	NMC0795	**NEIS0795**	hypothetical protein
NMBG2136_0798	NMBH4476_1314	NMA1073	**NMC0806**	nca	hypothetical protein
NMBG2136_0794	NMBH4476_1319	**NMA1078**	NMC0805	nca	hypothetical protein
NMBG2136_1836	NMBH4476_1885	**NMA0505**	NMC1918	**NEIS1918**	inner membrane transport protein
NMBG2136_1817	**NMBH4476_1867**	NMA0527	NMC1900	**NEIS1900**	lacto-N-neotetraose biosynthesis glycosyl transferase (lgtE)
NMBG2136_2028	**NMBH4476_2080**	NMA0225	NMC2118	**NEIS2118**	phospholipase, patatin family
-	-	-	**NMC0785**	nca	pseudogene
**NMBG2136_1630**	NMBH4476_0463	NMA2015	NMC1679	**NEIS1679**	putative 2-oxoglutarate/malate transporter
**NMBG2136_1820**	(NMBH4476_1869)	(NMA0524)	(NMC1902)	nca	putative glycosyl transferase
-	-	**NMA1058**	-	nca	putative glycosyl transferase (pseudogene)
**D. Synonymous and non-synonymous amino acid changes**					
**NMBG2136_1903 ^**	NMBH4476_1948	NMA0430	NMC1987	**NEIS1987**	ATP-dependent RNA helicase (hrpA)
**NMBG2136_0602**	NMBH4476_1533	NMA2115	NMC0599	**NEIS0599**	conserved hypothetical protein
NMBG2136_0948	**NMBH4476_1198**	NMA1174	NMC0957	**NEIS0957**	Flavodoxin-like fold family protein
**NMBG2136_0869**	NMBH4476_1488	NMA0905	NMC0870	nca	host specificity protein J / IgA-specific serine endopeptidase
NMBG2136_1893	NMBH4476_1938 *	NMA0440 *	**NMC1978**	**NEIS1978**	(nlpC) p60 family protein
**NMBG2136_0586**	NMBH4476_1542	-	NMC0586	**NEIS0586**	hypothetical protein
**NMBG2136_0589**	NMBH4476_1541	-	NMC0591	**NEIS0591**	hypothetical protein
**NMBG2136_0272**	**NMBH4476_0266**	NMA2214	NMC1767	**NEIS0785**	hypothetical protein
NMBG2136_0274 *	NMBH4476_0268	**NMA2214**	NMC0832	nca	hypothetical protein
**NMBG2136_0623**	**NMBH4476_1513 ^**	NMA0876	NMC0625	**NEIS0625**	hypothetical protein
NMBG2136_0368	**NMBH4476_1793**	NMA0607	NMC0369	nca	hypothetical protein
**NMBG2136_0543 ^**	NMBH4476_1585	NMA0810	NMC0548	**NEIS0548**	hypothetical protein
**NMBG2136_0662 ^**	NMBH4476_1474	NMA0921	NMC0666	**NEIS0666**	hypothetical protein
NMBG2136_0820	**NMBH4476_1292 ^**	NMA1641	NMC1340	nca	hypothetical protein
NMBG2136_1296	**NMBH4476_0860 ^**	NMA0458	NMC1340	nca	hypothetical protein

NMBG2136_0303	**NMBH4476_1858 ^**	NMA0537 *	NMC0306	**NEIS0306**	hypothetical protein
**NMBG2136_1304 ^**	NMBH4476_0811	NMA1626	NMC0527	nca	iron-regulated protein (frpA)
NMBG2136_1817	**NMBH4476_1868**	NMA0525	NMC1901	**NEIS1901**	lacto-N-neotetraose biosynthesis glycosyl transferase (lgtB)
NMBG2136_0361	**NMBH4476_1801**	NMA0600	NMC0361	**NEIS0361**	modulator of drug activity B
NMBG2136_1917	**NMBH4476_1961 ^**	NMA0422	NMC1997	**NEIS1997**	pantetheine-phosphate adenylyltransferase (coaD)
-	**NMBH4476_0626**	-	-	nca	pseudogene
-	**NMBH4476_0496 ^**	-	-	nca	pseudogene
-	**NMBH4476_1855 ^**	-	-	nca	pseudogene
**NMBG2136_0779 ^**	-	-	-	nca	pseudogene
-	-	**NMA0315**	-	nca	putative MafB alternative C-terminus (fragment)
**-**	**-**	**NMA0319**	**-**	nca	putative MafB alternative C-terminus (fragment)
**-**	**-**	**NMA0273A**	**-**	nca	putative membrane protein (pseudogene)
NMBG2136_1188	NMBH4476_0932 *	NMA1491	**NMC1214**	**NEIS1214**	transcription-repair coupling factor (mfd)
**E. Assembly failure due to long repeat tract**					
NMBG2136_1893	NMBH4476_1938 *	**NMA0440**	NMC1978 *	**NEIS1978**	(nlpC) p60 family protein
**NMBG2136_1714**	NMBH4476_0383	NMA2099	NMC1778	**NEIS1778**	aldose 1-epimerase
NMBG2136_1785	NMBH4476_0308	NMA2174	**NMC1858**	**NEIS1858**	conserved hypothetical protein
**NMBG2136_0927**	NMBH4476_1217	NMA1150	NMC0932	**NEIS0932**	dihydrolipoyllysine-residue succinyltransferase (sucB)
NMBG2136_1820	NMBH4476_1869	**NMA0832**	**NMC0568**	**NEIS0568**	glycosyl transferase
**NMBG2136_2007**	(NMBH4476_2058)	-	(NMC2091)	nca	hypothetical protein
**NMBG2136_1361**	NMBH4476_0752	(NMA0291)	(NMC0021)	nca	hypothetical protein
-	-	NMA1723	**NMC1452**	**NEIS1452**	hypothetical protein
NMBG2136_0520	NMBH4476_0489	**NMA0784**	NMC1653	**NEIS1653**	membrane protein
**NMBG2136_0282**	**NMBH4476_0276**	NMA2206	**NMC0276**	**NEIS0276**	peptidyl-prolyl cis-trans isomerase (surA) / rotamase
NMBG2136_1610	NMBH4476_0484	**NMA1996**	NMC1658	**NEIS1658**	periplasmic type I secretion system protein | hemoysin D
NMBG2136_0010	**NMBH4476_0010**	NMA0257	NMC2148	**NEIS2148**	phosphoglycerate kinase
**NMBG2136_0563**	-	-	-	nca	pseudogene
**NMBG2136_1786**	-	-	-	nca	pseudogene
-	**NMBH4476_0307**	-	-	nca	pseudogene
-	**NMBH4476_0843**	-	-	nca	pseudogene
-	**NMBH4476_1566**	-	-	nca	pseudogene
**NMBG2136_1404**	**NMBH4476_0706**	NMA1733	NMC1462	**NEIS1462**	putative lipoprotein
NMBG2136_1188	**NMBH4476_0932**	NMA1491	NMC1214 *	**NEIS1214**	transcription-repair coupling factor (mfd)
NMBG2136_0407	**NMBH4476_1758**	NMA0650	NMC0408 *	**NEIS0408**	type IV pilus secretin (pilQ)
**F. Locus is paralogous in the genome**					
**NMBG2136_0915;<br>NMBG2136_1845**	**NMBH4476_1229;<br>NMBH4476_1894**	**NMA1138;<br>NMA0495**	**NMC0920;<br>NMC1928**	**NEIS0920;<br>NEIS1928**	50S ribosomal protein subunit L31 (rpmE)
**NMBG2136_0914;<br>NMBG2136_0160**	**NMBH4476_1230;<br>NMBH4476_0161**	**NMA1137;<br>NMA0107**	**NMC0919;<br>NMC0154**	**NEIS0919;<br>NEIS0154**	50S ribosomal protein subunit L36 (rpmJ)
NMBG2136_0942;<br>NMBG2136_1614	NMBH4476_0479;<br>NMBH4476_1204;<br>NMBH4476_1694	NMA1168;<br>NMA2000	**NMC0951;<br>NMC1662**	**NEIS0951;<br>NEIS1662**	conserved hypothetical protein
NMBG2136_0121	NMBH4476_0121	**NMA0149;<br>NMA0134**	NMC0116	**NEIS0116**	elongation factor TU (tufA2)
-	**NMBH4476_1709;<br>NMBH4476_1706**	-	-	nca	hypothetical protein
NMBG2136_0604	NMBH4476_1532	NMA0036	**NMC1803;<br>NMC0223**	nca	hypothetical protein
-	**NMBH4476_1692;<br>NMBH4476_0736**	NMA0594	NMC1424	nca	hypothetical protein
NMBG2136_0607	NMBH4476_1538;<br>NMBH4476_1529	NMA0856;<br>NMA0858;<br>NMA0857	**NMC0595;<br>NMC0608;**<br>(NMC0594)	**NEIS0595;<br>NEIS0608**	hypothetical protein

NMBG2136_0967	NMBH4476_1082	**NMA1193; NMA1859**	NMC0973	**NEIS0973**	hypothetical protein
NMBG2136_1605	NMBH4476_0489	**NMA1989**	NMC1653	**NEIS1653**	hypothetical protein
**NMBG2136_1730; NMBG2136_1733;** NMBG2136_1729	**NMBH4476_0361; NMBH4476_0367**	NMA2118	NMC1796; NMC0600; NMC1795	**NEIS1796; NEIS0600; NEIS1795**	hypothetical protein
NMBG2136_0023	**NMBH4476_0593; NMBH4476_0686**	NMA1793	NMC0030	nca	hypothetical protein
NMBG2136_1358	NMBH4476_0755	NMA0862	**NMC1403; NMC1551**	nca	hypothetical protein; (opaA)
NMBG2136_1658	NMBH4476_0591; NMBH4476_0435	(NMA0862)	**NMC1719; NMC0903**	**NEIS1719**	Opa1800 outer membrane protein (opaJ); hypothetical protein (opaD)
NMBG2136_0787; NMBG2136_0518	**NMBH4476_1333; NMBH4476_1608**	NMA1066; NMA1066	NMC0795; NMC0524	**NEIS0795; NEIS0524**	peptidase, C39 family
NMBG2136_0795	**NMBH4476_1325; NMBH4476_1315**	NMA1047	NMC0802	nca	putative lipoprotein

hypothetical protein: a protein whose existence has been predicted, but for which there is no experimental evidence that it is expressed in vivo

putative protein: a region of genomic DNA is predicted to contain a protein-coding gene, ab initio gene finding with extrinsic evidence that the gene is functional

pseudogene: dysfunctional genes that have lost their protein-coding ability or are otherwise no longer expressed

[#] Definition of locus may vary between finished genomes depending on the gene finding algorithim and/or annotation software used; listed definition is specific to the genome listed.

* The same locus, in two separate genomes, has different sequence or assembly issue.

^ Indicates that that the sequence discrepancy was caused by an insertion/deletion.

nca NEIS locus tag identifier currently not assigned, however locus is being curated for future addition to the sequence definition database.
