# Supplementary material for: A gene-by-gene population genomics platform: de novo assembly, annotation and genealogical analysis of 108 representative Neisseria meningitidis genomes
Source: BMC Genomics. 2014 Dec 18;15(1):1138. doi: 10.1186/1471-2164-15-1138 (PMC4377854; doi:10.1186/1471-2164-15-1138)
Supplement: Supplementary file 3 — Additional file 3: Table S3: Sequence discrepancy categories. (PDF 177 KB) [file 12864_2014_6881_MOESM3_ESM.pdf]

**Additional Table 3** Sequence discrepancy categories

| **single nucleotide sequence change per allele resulting in** | **number of alleles affected per isolate** | | | |
|---|---|---|---|---|
| | **Z2491** | **FAM18** | **H44/76** | **G2136** |
| non-synonymous amino acid change | 8 | 5 | 4 | 3 |
| synonymous amino acid change | 3 | 3 | 5 | 7 |
| **multiple nucleotide sequence change per allele resulting in** | **number of alleles affected per isolate** | | | |
| | **Z2491** | **FAM18** | **H44/76** | **G2136** |
| non-synonymous amino acid change | 4 | 1 | 1 | 3 |
| synonymous amino acid change | 1 | 0 | 0 | 0 |
| non & synonymous amino acid change | 4 | 2 | 1 | 0 |
| **insertion/deletion change per allele resulting in** | **number of alleles affected per isolate** | | | |
| | **Z2491** | **FAM18** | **H44/76** | **G2136** |
| single insertion/deletion | 0 | 0 | 3 | 3 |
| double insertion/deletion | 0 | 0 | 3 | 3 |
